# Von Noorden's Dietary in Mucous Colitis

**Published:** 1908-02-15

**Authors:** 


### VON NOORDEN'S DIETARY IN MUCOUS COLITIS.

There are two Schools of Dietists as regards the treatment of patients suffering from mucous colitis—the von Noorden School and the French School. These are diametrically opposed to one another in principle; nevertheless, both have succeeded in giving relief to patients with the disease in question. The French School regard mucous colitis as essentially an inflammation of the colon; and all their efforts are devoted to devising dietaries that leave the least possible fæcal residue, the idea being that the less the material that scrapes the surface of the inflamed bowel the more likely is the inflammation to diminish or even disappear. It is, however, of the von Noorden School that we would speak here, in response to letters that we have received from our readers. The gist of von Noorden's dietetic treatment of the disease is to administer daily as much as possible of all kinds of foods that contain an abundance of cellulose. Cellulose is readily digested by herbivorous animals, but the juices of the human alimentary canal leave it for the most part unaltered. Hence, upon a diet containing quantities of cellulose there is a bulky fæcal residue, and it is in virtue of this that von Noorden advocates it in mucous colitis. The idea underlying this seems to be that constipation is an essential factor in the disease, and that steps which will naturally overcome the constipation will ameliorate the other symptoms also. Hence, von Noorden advises the patients to eat whole-meal bread with every meal; to take plenty of porridge to breakfast, to avoid potatoes, pastry, white bread, and, indeed, all forms of carbohydrate in which the percentage of cellulose is small, replacing these by other forms, such as green vegetables of all kinds, fruit such as apples, bananas, and so forth, and conserves such as marmalade, rather than jellies or jams containing less cellulose. It is clear that, provided the cellulose principle is borne in mind, the dietary can be greatly varied according to the idiosyncrasies of different patients. Some will take fresh fruit to every meal, some only at breakfast, others only when going to bed, and so on. Some will take porridge twice a day, others only in the morning; but the general principle is the same in all. It is right to mention that in practice this line of treatment succeeds less often than it fails. It is chiefly in the milder cases that it is of most benefit. In severer cases the pains and tenderness along the course of the colon may be so much increased that the patients refuse to go on with the treatment. In early cases, however, or in those in whom the constipation is soon overcome by the bulkiness of the fæcal residues, the treatment is good. It is in those cases in which constipation persists as before, whilst the colon is at the same time forced to contain the increased bulk of fæces, that it fails.

